# The impact of public lighting improvement on crimes and incivilities reduction in Chile

**DOI:** 10.3389/fsoc.2025.1541359

**Published:** 2025-03-20

**Authors:** Pablo Cadena-Urzúa, Javier Guardiola, Francisco Montes

**Affiliations:** ^1^Doctoral Programme in Law, Political Science and Criminology, Department of Criminal Law, Universitat de València, València, Spain; ^2^University Research Institute of Criminology and Criminal Science, Universitat de València, València, Spain; ^3^Department of Statistics and Operational Research, Universitat de València, Burjassot, Spain

**Keywords:** public space crime, incivilities, public lighting, situational prevention, propensity score methods

## Abstract

The objective of this study is to evaluate the impact of the Public Lighting Replacement Programme on crimes and incivilities reduction in various municipalities in Chile. A quantitative approach was employed, using stratification and matching techniques to simulate randomized trials based on observational data. Data were extracted from governmental sources and statistics for the period 2015–2019, covering 101 municipalities where lighting improvements were implemented and 244 municipalities that were not treated. The main results suggest that improvements in public lighting could be associated with a reduction in certain crimes, such as surprise robbery, and various incivilities, such as street vending and alcohol consumption in public spaces. However, some crimes, such as violent robberies, did not show significant decreases following the intervention. In conclusion, modernizing public lighting can contribute to the prevention of both crime and incivilities, but its impact is heterogeneous. Unobserved factors, underreporting, and implementation variations may influence results. Complementary measures, such as targeted patrolling, are essential to address violent crime and persistent incivilities. This study underscores the need for integrated urban security strategies to enhance public safety in Chile.

## 1 Introduction

The relationship between improvements in public lighting and its influence on crime has been a growing topic of interest for both policymakers and researchers in the field of urban security (Perkins et al., 2015; Arvate et al., 2018; Kang and Yeom, 2019; Mihale-Wilson et al., 2019; Davies and Farrington, 2020; Struyf, 2020). However, to date, there is a significant gap in the Chilean context regarding the understanding of how the replacement and renewal of lighting infrastructure in public spaces may affect crime incidence in local communities.

By contrast, in other geographical contexts, there is scientific literature on this issue. For example, Kaplan and Chalfin (2022) analyses the extent to which street lighting improvements affect daily night-time activities and concludes that enhancing public lighting can help control crime and improve community well-being. In Chalfin et al. (2022), a study on street lighting in New York, the results suggest that a tactical deployment of lighting can control crime through a deterrent effect that leads to a reduction in outdoor night-time crimes. Finally, Welsh and Farrington (2008) conduct a study on the effect of improved public lighting on crime, reviewing publications related to the United States and Great Britain. They obtain two interesting findings: on the one hand, they observe a significant reduction in crime, but this reduction was not greater for night-time crimes than for daytime ones, leading the authors to suggest, “that a theory of street lighting focusing on its role in increasing community pride and informal social control may be more plausible than a theory focusing on increased surveillance and increased deterrence.”

The Public Lighting Replacement Programme 200,000 Lights, promoted by the Ministry of Energy and the Undersecretary of Energy of Chile, has emerged as a key initiative in modernizing lighting infrastructure nationwide. This programme, which aimed not only to improve energy efficiency but also to address public safety concerns, represents a relevant case study for exploring the impacts of public lighting improvements on situational crime prevention.

Crime in public spaces, a constant concern in Chilean communities, has been the subject of various investigations and intervention policies. However, the specific connection between the quality of public lighting and the occurrence of crimes in these settings has received limited attention in academic and scientific literature, leaving a gap in our understanding of the factors that influence urban safety. An exception is the work of Domínguez and Asahi (2023), in which the authors study the effect of sunlight on crime, particularly during the switch to daylight saving time, finding a decrease in thefts when the daily period of sunlight is longer. The economic impact of crime is also relevant; in this regard, it is worth citing the book by Di Tella et al. (2010) which deals with this impact in Latin America.

The absence of studies, as far as we know, evaluating the impact of the aforementioned programme on crime incidence in Chilean municipalities underscore the need for scientific investigations addressing this issue. This research positions itself to fill this knowledge gap, providing empirical evidence on the effect of public lighting improvements on crimes and incivilities prevention and offering valuable insights for informing future urban safety policies and practices.

This study examines the possible relationship between the Public Lighting Replacement Program and the reduction of crimes and incivilities in various municipalities in Chile, estimating associations through propensity score matching techniques. While these techniques allow for approximating causal inferences in observational studies, the presence of unobserved factors prevents the establishment of definitive causality.

In Welsh and Farrington (2008), the authors cite two main theories explaining why improvements in public lighting may reduce crime. The first suggests that better lighting, which improves visibility and increases the number of people on the street, results in greater surveillance of potential offenders and consequently leads to greater deterrence. The second theory suggests that improving lighting requires community investment in the area, thereby increasing pride and cohesion within the community and enhancing informal social control. The first theory predicts a reduction in crime, especially during dark hours, while the second theory extends the reduction to both daytime and night-time crime. These premises justify our research.

To conduct our analysis, we will use data from governmental and public safety sources as well as specific information on the Public Lighting Replacement Programme. These data will allow us to conduct an empirical evaluation of the programme's impact on urban safety, providing a solid basis for our conclusions.

With the available data, we will estimate the effect of the public lighting replacement on crime occurrence in Chilean municipalities using two techniques derived from propensity score matching (Austin, 2009, 2011), namely stratification and matching.

In order to interpret our results, it is useful to employ criminological frameworks such as the broken windows theory (Wilson and Kelling, 1982), rational choice theory (Cornish and Clarke, 1986), and routine activity theory (Cohen and Felson, 1979). The first theory (which promoted zero-tolerance policies, much criticized today, but also enhanced studies on disorder and criminality and oriented crime prevention through environmental design) posits that visible signs of disorder, such as poor lighting, foster increased criminality by suggesting a lack of social control. The improvement of public lighting may have reinforced informal social control and community cohesion, reducing incivilities and certain minor crimes, such as surprise robberies and street vending. The significant reduction of these crimes and incivilities in the treated municipalities supports this theory, which has also been suggested by prior studies (Welsh and Farrington, 2008). Regarding the conjecture of Cornish and Clarke (1986), offenders make decisions based on a rational assessment of costs and benefits. By improving lighting, the perceived costs of committing crimes increase due to greater visibility and the likelihood of being apprehended. This principle explains the reduction of opportunistic crimes such as surprise robbery, as offenders may perceive higher risks in better-lit areas. As pointed out formally by Becker (1968), “optimal policies to combat illegal behavior are part of an optimal allocation of resources”. The assumption of Cohen and Felson (1979) also becomes relevant, as it proposes that crimes occur when a motivated offender, a potential victim, and a lack of effective guardians converge. Improving lighting increases visibility and foot traffic, deterring offenders by enhancing the perception of present guardians.

The article is structured into six sections. In Section 2, we present the main characteristics of the variables and data. Next, in Section 3, we describe the methodology based on the propensity score measure and specify the two techniques that will allow us to empirically analyse the effect of lighting renewal on public space crime in Chilean municipalities. In Section 4, we discuss the main findings of this research. The relationship of our results to the published literature, as well as their limitations and future research directions, are presented in Section 5. A Section 6 with Conclusions and Limitations of our study, and the Bibliography conclude the article.

## 2 Data and variables

The data used in this study came from five government sources: the Centre for the Study and Analysis of Crime (CEAD), the Undersecretariat of Energy, the National Statistics Institute (INE), the National Congress Library (BCN) and the National Municipal Information System (SINIM). In order to ensure the coherence and compatibility of the information, a standardization and validation process was carried out, which included the standardization of measurement units, the consolidation of crime and incivility categories and the harmonization of socio-economic variables.

Data integration was done using a structured approach. First, key variables were identified for each source to ensure comparability in time and space. For example, CEAD records provided data on crimes committed in public spaces, while the SINIM and INE databases complemented the information with socio-demographic and economic indicators at the municipal level.

Cross-validation criteria were then applied to detect inconsistencies and biases in the reports, particularly in the reporting of crimes and incivilities. To mitigate the effects of underreporting, trends in citizen complaints were compared with official records, and consistency thresholds were set to minimize bias in the estimation of the impact of the intervention.

Finally, the extracted variables were grouped into three categories: (1) indicators of crimes and incivilities, (2) socio-economic characteristics of the municipality, and (3) data on the implementation of the public lighting programme.

We selected the municipality as the spatial unit of analysis because it is the political and administrative division used in the country. Moreover, we analyzed the years 2015–2019 for two reasons: first, 2015 serves as the pre-treatment year, prior to the implementation of the lighting replacement programme; second, the replacement of streetlights was carried out until 2018, so 2019 is considered the post-treatment year. Furthermore, the restrictions imposed during the global COVID-19 pandemic affected the normal pattern of crime, making subsequent years unsuitable, as they would distort the intended comparison.

In summary, we collected data on public space crimes and incivilities committed in 101 municipalities in Chile before and after the public lighting improvement. This is supplemented with equivalent data for the same period from the 244 municipalities that did not upgrade their lighting. The lighting upgrades were carried out in 12 municipalities in 2016, 72 municipalities in 2017, and 17 municipalities in 2018, covering the three central years of the 2015–2019 five-year period. This implies that in 2015, all municipalities had the old lighting system, and by 2019, the upgrade was completed in the 101 municipalities that underwent the change.

The variables related to crimes and incivilities are presented in Table 1. All these variables refer to activities occurring in public spaces and may therefore be influenced by lighting conditions.

**Table 1:** Variables.

**Crimes**	**Incivilities**
Theft	Street vending
Violent robbery	Alcohol consumption
Surprise robbery	Property damage
Vehicle theft	Public disorder
	Public drunkenness
	Public brawls

Additionally, socio-economic information for the municipalities is available as shown in Table 2. Some of these variables deserve a more detailed explanation. The *Share of permanent own-source revenues in total income* refers to the portion of internally generated municipal revenues over total revenues (e.g. land taxes, municipal patents, among others), and the *Share of community services sector in total expenditure* is the portion of municipal expenditures dedicated to community services, such as social and recreational programmes. The *CASEN poverty index* is the municipal poverty index derived from The National Socioeconomic Characterization Survey (CAracterización SocioEconómica Nacional), carried out by the Ministry of Social Development and Family. The *Municipal primary health care coverage* measures the percentage of the population that has access to primary health care services in the municipality. The *Primary health care activity index* assesses the efficiency and productivity of health centers in terms of the number of services provided, such as medical consultations, check-ups, and home visits. The choice of these socio-economic variables is justified by their influence on crime and perceptions of security (Bellitto and Coccia1, 2018; Saravanan et al., 2021; Cadena-Urzúa et al., 2022).

**Table 2:** Socio-economic variables.

**Variables**
Population density
Female-headed households (%)
Infant mortality rate
Population belonging to an ethnic group (%)
Foreign population (%)
Share of permanent own-source revenues in total income (%)
Share of community services sector in total expenditure (%)
Dropout rate in primary education in municipal establishments (%)
Dropout rate in secondary education in municipal establishments (%)
Municipal primary healthcare coverage (%)
Primary healthcare activity index (%)
CASEN poverty index (%)
Community organizations per 100,000 inhabitants
Total municipal area (Km^2^)
Total area of existing parks in the municipality (m^2^)
Green areas (m^2^) with maintenance per inhabitant
Distance in km to the regional capital
Rural population (%)
Overcrowded housing (%)
Employment rate
Energy consumption (kWh) as a proxy for economic activity

## 3 Models for estimating the effect based on a propensity measure

Unlike a Randomized Controlled Trial (RCT), where the items to be treated are randomly selected to eliminate potential biases when measuring the effect of the treatment, our data correspond to what is known as an observational study, whose objective (Cochran, 1965) is “to elucidate cause-and-effect relationships”, but in which “it is not feasible to use controlled experimentation, in the sense of being able to impose the procedures or treatments whose effects one wishes to discover, or to assign subjects at random to different procedures”. In both contexts, the aim is to understand the effect that a particular treatment has.

The method described here seeks to emulate randomized trials by selecting controls among the untreated items. To achieve this, a propensity measure is calculated, which, as defined by Rosenbaum and Rubin (1983, 1985), is the conditional probability of assignment to a given treatment, given a vector of observed covariates. A logistic model is typically used to obtain this measure. Once obtained, each treated item (case) is matched with one or more untreated items (controls) based on the proximity of their respective propensity scores (Apel and Sweeten, 2010). This simulates a control system similar to that of randomized trials, allowing the estimation of the treatment effect–in our case, the improvement due to lighting–using various methods (Chatton et al., 2020), of which we have chosen two: stratification and matching. The selection of both methods is based on their ability to improve the balance between treated and untreated groups, reducing selection biases without requiring rigid parametric specifications, and providing a parsimonious compromise between simple models, such as OLS, and more sophisticated models such as two way fixed effects regression.

### 3.1 Stratification

This method involves stratifying the propensity score into a set of classes containing approximately the same number of elements in the sample. Typically, division into 5 strata (quintiles) is used, as justified in the work of Cochran (1968), though we will also use a division into 10 strata (deciles). The estimation of the average treatment effect on the treated (ATT) is obtained as a weighted average of the mean effect in each stratum, expressed as:


$$ATT=\sum_{s}\frac{N_{s}}{N}({\bar{Y}}_{T=1,s}-{\bar{Y}}_{T=0,s}),$$

where *N*_*s*_ and *N* are the number of elements in stratum *s* and the total number of elements in the sample, respectively, and Ȳ_*T* = 1, *s*_ and Ȳ_*T* = 0, *s*_ are the mean number of crimes in stratum *s* for the treated (*T* = 1) and untreated (*T* = 0) municipalities.

It should be noted that the above expression does not provide a standard error for the estimated effect, and it is therefore not possible to calculate an associated p-value. This issue can be addressed by conducting *R*−1 random permutations of the treatment variable, estimating ATT for each permutation, and obtaining the percentile of the original estimate among the *R* values.

### 3.2 Matching

This second method is based on matching each treated municipality with one or more untreated municipalities. Several methods can be used to achieve this. Here, we will use the method described in the MatchIt package (Ho et al., 2011) for R software (R Core Team, 2023), which the authors call optimal, as it minimizes the sum of absolute distances between pairs in the matched sample. This method offers two options: one where the ratio of controls matched to each case is fixed beforehand, making it the same for all cases without necessarily exhausting all controls, and another where no such limitation is applied–this is called the full option, where all available controls are used and match sizes can vary.

The effect estimation is obtained using the G-computation method, which requires fitting a regression model to the outcome of interest—in our case, crimes and incivilities. The model can be a linear regression that uses not the original dataset but the one resulting from the matching process, as suggested by Ho et al. (2007), to reduce the dependence of the effect estimation on the correct specification of the model. It is also advisable to include the covariates used to obtain the propensity score, as this improves the precision of the effect estimation. The model may also include the interaction between the treatment and the covariates (Greifer, 2024).

The R packages MatchIt, as mentioned earlier, and marginaleffects (Arel-Bundock, 2024) were used to apply the described methods.

### 3.3 Model

The analysis using stratification was carried out, as mentioned earlier, with 5 and 10 strata, respectively. For matching, three options of the optimal method were considered: associating 3 and 4 controls with each case and using all available controls (full). This technique requires obtaining the propensity score first, which, in our case, represents the probabilities of belonging to the group of treated municipalities derived from a logistic regression involving the socio-economic variables listed in Table 2.

All these variables have been included in the model in order to control for structural differences between municipalities and to reduce possible biases in treatment assignment. Covariate balancing was checked by comparing distributions before and after matching, ensuring that matched municipalities had similar characteristics before the intervention.


      logit(treatment) ~
      Population_Density + Female_Headed_Households +
      Infant_Mortality + Ethnic_Population + Foreign_Population + IPP +
      Community_Service_Expenditure +  Primary_Education_Dropout +
      Secondary_Education_Dropout + Municipal_Healthcare_Coverage +
      Primary_Healthcare_Activity + CASEN_Poverty_Index +
      Community_Organizations + Municipal_Area + Park_Area +
      Green_Areas + Distance_to_Regional_Capital + Rural_Population +
      Overcrowded_Housing + Employment + Energy_kwh


Recall that G-computation requires a regression model of the outcome variable on a given set of covariates. The model used was:


      Y ~ treatment*(propensity_score +
      Population_Density + Female_Headed_Households +
      Infant_Mortality + Ethnic_Population + Foreign_Population + IPP +
      Community_Service_Expenditure +  Primary_Education_Dropout +
      Secondary_Education_Dropout + Municipal_Healthcare_Coverage +
      Primary_Healthcare_Activity + CASEN_Poverty_Index +
      Community_Organizations + Municipal_Area + Park_Area +
      Green_Areas + Distance_to_Regional_Capital + Rural_Population +
      Overcrowded_Housing + Employment + Energy_kwh)


where *Y* is one of the crimes or incivilities. Following Ho et al. (2007) and Greifer (2024), a linear regression was used with the same covariates as the logistic model plus the propensity score. The model also includes the interaction of the treatment with all covariates.

## 4 Results and findings

Table 3 shows the results of estimating the effect that the installation of new lighting had in the municipalities, specifically the reduction in crimes and incivilities listed in Table 1. To correct for population effects, we worked not with absolute frequencies but with the ratio of crimes per 10,000 inhabitants.

**Table 3:** Effects estimation for different methods.

	**Stratification**				**Matching**					
	**10 strata**		**5 strata**		**Optimal ratio 3**		**Optimal ratio 4**		**Optimal all**	
**Crime/ incivility**	**Estimate**	***p*** **-value**	**Estimate**	***p*** **-value**	**Estimate**	***p*** **-value**	**Estimate**	***p*** **-value**	**Estimate**	***p*** **-value**
Thefts	−2.71	0.118	−3.34	0.082	−3.01	0.117	−3.25	0.089	−3.56	0.078
Violent robbery	1.70	0.978	1.11	0.900	0.99	0.194	1.04	0.182	0.93	0.196
Surprise robbery	−1.67	0.002	−1.81	0.002	−1.95	0.000	−1.95	0.000	−2.50	0.000
Vehicle theft	−0.18	0.444	−1.33	0.136	0.13	0.879	0.17	0.852	0.34	0.735
Street vending	−2.93	0.016	−3.14	0.006	−2.39	0.010	−2.31	0.030	−2.77	0.003
Alcohol consumption	−14.47	0.022	−17.37	0.014	−13.10	0.076	−12.95	0.085	−20.50	0.008
Damages	−2.01	0.118	−2.38	0.084	−2.16	0.145	−2.49	0.092	−2.62	0.105
Disturbances	−0.33	0.044	−0.31	0.054	−0.62	0.002	−0.67	0.001	−0.95	0.000
Public brawls	−0.30	0.042	−0.26	0.088	−0.30	0.080	−0.35	0.043	−0.35	0.048
Aggregated crimes	−2.86	0.234	−5.37	0.074	−3.83	0.167	−4.00	0.144	−4.79	0.135
Aggregated incivilities	−24.05	0.010	−27.17	0.008	−19.26	0.050	−19.47	0.052	−29.85	0.003

As shown in Table 3, those effects deemed significant are highlighted in gray, with darker gray indicating significance at the 5% level and lighter gray indicating significance at the 10% level. While these results will be discussed in detail later, one aspect merits immediate attention: the quantitative and qualitative coherence of the findings. The former is evident in the similar effect estimates across all employed methods and their consistent sign. Regarding the latter, where significance is noted at the 5% level, it also remains consistent across all methods, at most shifting to the 10% level but never exceeding it.

The effects observed are negative for all crimes and incivilities, indicating a decrease in their incidence, except for violent robberies, which appear to increase slightly with the improvement of public lighting across all techniques employed.

As highlighted in Table 3, not all effects are significant. Among the crimes, only surprise robberies show a significant reduction. Conversely, only two incivilities, damages and drunkenness, show no changes; the remaining four exhibit reductions, resulting in a significant effect when aggregated, though not for the aggregation of crimes.

It is interesting to assess the average percentage reduction that the significant effects entail. Table 4 presents these figures, referencing the average ratios of crimes and incivilities from the year 2015, the year prior to the initiation of the lighting improvement programme. The table shows the percentage reduction for various techniques for crimes and incivilities that exhibit significant effects. The averages are calculated for the municipalities involved in the lighting improvement programme.

**Table 4:** Effect on the percentage reduction of crimes and incivilities ratios per 10,000 inhabitants.

		**Stratification**		**Optimal matching**		
**Crime/incivility**	**Average 2015**	**10 strata**	**5 strata**	**Ratio 3**	**Ratio 4**	**All**
Surprise robbery	8.38	19.93	21.60	23.28	23.28	29.84
Street vending	5.68	51.59	55.29	42.08	40.67	48.77
Alcohol consumption	67.26	21.52	25.83	19.48	19.25	30.48
Disturbances	1.63	20.19	18.97	37.93	40.99	58.12
Public brawls	2.37	12.66	10.98	12.66	14.77	14.77

As previously noted, the differences in estimated effects are not pronounced across the various techniques for the same crime or incivility, and this holds true for the percentages as well. However, significant differences do exist between different crimes and incivilities, ranging from a 10.98% reduction in public brawls using the estimate with 5 strata to a 58.12% reduction in disturbances, estimated using optimal matching across the entire sample. Street vending exhibits the largest overall reductions, with none falling below 40%. Although not presented in the table, which refers only to isolated crimes or incivilities, the reduction in aggregated incivilities varied between 9.52% and 14.76%, depending on the technique.

## 5 Discussion

The findings of this study provide correlational evidence on the possible relationship between improved public lighting and the reduction of crimes and incivilities in the intervened municipalities, in line with the existing literature.

Several prior studies, such as that by Kaplan and Chalfin (2022), have emphasized the relationship between improved lighting and the reduction of nighttime criminal activities in public spaces. In this regard, our findings align with these investigations, as crimes such as surprise robbery significantly decreased following the implementation of the lighting replacement programme. This pattern is also observed in previous studies conducted in various cities and countries, such as New York (Chalfin et al., 2022) and Great Britain (Welsh and Farrington, 2008).

When comparing our results with the literature, it is noteworthy that while the improvement of public lighting contributes to the reduction of certain crimes, not all types of criminal activity were similarly affected. For instance, as in the study by Welsh and Farrington (2008), we found that violent crimes did not experience significant reductions following the lighting enhancement, suggesting that merely increasing visibility is insufficient to deter more aggressive behaviors.

Moreover, our results confirm the findings of Domínguez and Asahi (2023), who discovered that increased exposure to light reduces certain opportunistic crimes, such as surprise robberies, which was a key finding in this research. The slight increase in violent robberies is not statistically significant, further research is needed before definitive conclusions can be drawn. Future research could investigate whether increased lighting facilitates victim resistance, thereby changing the dynamics of crime, and assess other contextual factors such as changes in surveillance patterns or offender adaptation. This hypothesis requires further empirical support to better understand its implication.

However, the observed variation in the reduction of incivilities (such as street vending and alcohol consumption) indicates that the effects of lighting improvement on social behavior are heterogeneous.

## 6 Conclusions and limitations

Although the results are promising, the association between improved public lighting and the reduction of crimes and incivilities was not homogeneous across all municipalities. For example, violent robberies did not experience a significant reduction, indicating that additional interventions beyond improving lighting infrastructure are necessary to address such crimes. Future research should examine whether other factors, such as targeted police patrols and investment in community programmes, could enhance the deterrent effects of public lighting. In conclusion, our findings are consistent with criminological theories and reinforce the importance of urban infrastructure improvements as a tool in situational crime prevention.

Despite efforts to minimize biases and control confounding factors, this study has several limitations inherent to its observational design. Firstly, the inability to control for all unobserved variables prevents the establishment of a definitive causal relationship. Factors such as specific police patrolling strategies, concurrent local policies, and socioeconomic dynamics may have influenced the results. Secondly, reliance on police records and citizen reports introduces the risk of underreporting, the extent of which may vary across municipalities and crime types, potentially affecting the estimation of the intervention's actual impact. Finally, the analysis does not distinguish between the installation of new lighting and the improvement of existing systems, which could introduce heterogeneity in the observed effects.

## Data Availability

Publicly available datasets were analyzed in this study. The data used in this analysis can be provided on request to the authors.
